# The characteristic of *corvus pectoralis*’s complete mitochondrial genome and phylogeny analysis

**DOI:** 10.1080/23802359.2019.1676674

**Published:** 2019-10-11

**Authors:** Tian Huang, Libo Zhou, Zhenggang Xu

**Affiliations:** a College of Information and Electronic Engineering, Hunan City University, YiYang, China;; b Hunan Engineering Research Center for Internet of Animals, Changsha, China;; c Key Laboratory of Forestry Remote Sensing Based Big Data & Ecological Security for Hunan Province, Central South University of Forestry and Technology, Changsha, China

**Keywords:** *Corvus pectoralis*, Passeriformes, mitochondrial genome, phylogeny

## Abstract

The Collared Crow (*Corvus pectoralis*), in the order Passeriformes, it widely distributed in large areas encompassing China and northern Vietnam. It is a vulnerable bird that is of international concern. In this study, we first sequenced and described the complete mitochondrial genome and phylogeny of *C. pectoralis*. The results showed that the whole genome of *C. pectoralis* was 16,857 bp long and contains 13 PCGs, 2 ribosomal RNA genes, 23 transfer RNA genes, and 1 loop region. The overall base composition of the mitochondrial DNA was 31.13% for A, 29.52% for C, 24.46% for T, and 14.89% for G, with a GC content of 44.41%. The phylogenetic tree showed that *C. pectoralis* was clustered with *C. brachyrhynchos* and then together with other two crows in family Passeriformes. This information will be useful in the current understanding of the phylogeny and evolution of Passeriformes.

*Corvus pectoralis*, which belongs to the Passeriformes, widely distributed in China and northern Vietnam (John et al. 2015). It can be easily found in open areas where trees are scattered, especially near waters. In recent years, due to the intensification of human agriculture and the consequent overuse of pesticides and rodenticides, the reduction in prey has led to a decline in the population of the species within its range (Londei 2013), and it has been listed as a vulnerable group (VU) on the IUCN Red List of Threated Species (IUCN, 2019). Despite this, genetic information of *C. pectoralis* is quite limited. Haring et al. (2012) explored the appearance variation of the genus *Corvus* species based on the DNA sequence of the mitochondrial control region, and did not study the evolutionary classification of this species based on the complete mitochondrial genome. Therefore, it’s necessary to sequence the complete mitochondrial genome of *C. pectoralis* to enhance our understanding of the phylogeny and evolution of Corvidae.

The whole mitochondrial DNA was extracted from muscle specimen with DNeasy Plant Mini kit (Qiagen, Valencia, CA) and specimen was collected from Dongting Lake and stored at Hunan Engineering Research Centre for Internet of Animals, China with accession number 20170801PC. The genomic DNA data were sequenced by Illumina Miseq platform (Illumina, San Diego, CA). The adapter and low quality reads were filtered out by NGS QC toolkit (Patel and Jain 2012). The genome was annotated using the MITOS online service (Bernt et al. 2013). Annotated PCGS were compared with other vertebrate species sequences and the method referred to Zhang’s addressing (Zhang et al. 2017). The complete mitochondrial genome of *C. pectoralis* has been submitted to the NCBI database with the accession number of MN310552. Phylogenetic tree among *C. pectoralis* and its related orders were presented using 13 PCGs by Neighbor-joining analyses in MEGA 7.0 with 1000 bootstrap replicates (Kumar et al. 2016).

The complete mitogenome of *C. pectoralis* is 16 857 bp long and contains 13 PCGs, 2 ribosomal RNA genes, 23 transfer RNA genes, and 1 loop region. This feature was similar to the typical mitogenome of other birds (Ren et al. 2016; Wang et al., 2016; Huang et al. 2018). The overall base composition is 31.13% A, 29.52% C, 24.46% T and 14.89% G. The average length of 13 PCGs genes is 875 bp. All the protein-coding genes use the initiation codon ATG except COI uses GTG, which is quite common in vertebrate mtDNA (Lin et al. 2018; Peng et al. 2019). All of the nodes were inferred with strong support by the NJ analysis. The phylogenetic tree showed that *C. pectoralis* clustered with *C. brachyrhynchos* and then together with other two crows in family Passeriformes (Figure 1). In all, the mitochondrial genome reported here would be useful in the current understanding of the phylogeny and evolution of Passeriformes. We must pay more attention on *Corvus* species for lacking genetic information.

**Figure 1.**
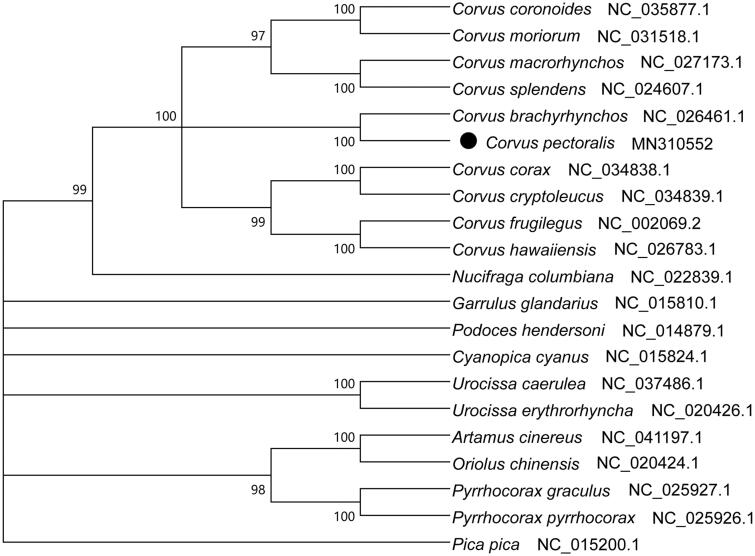
Phylogenetic tree of the relationships among Passeriformes and its related orders based on 13 PCGs.
